# Estimating Type 2 Diabetes Prevalence: A Model of Drug Consumption Data

**DOI:** 10.3390/pharmacy12010018

**Published:** 2024-01-22

**Authors:** Rita Oliveira, Matilde Monteiro-Soares, José Pedro Guerreiro, Rúben Pereira, António Teixeira-Rodrigues

**Affiliations:** 1 FP-BHS—Biomedical and Health Sciences Research Unit, FFP-I3ID—Instituto de Investigação, Inovação e Desenvolvimento, Faculdade de Ciências da Saúde, Universidade Fernando Pessoa, Rua Carlos da Maia 296, 4200-150 Porto, Portugal; 2 UCIBIO—Applied Molecular Biosciences Unit, MedTech, Laboratory of Pharmaceutical Technology, Department of Drug Sciences, Faculty of Pharmacy, University of Porto, Rua Jorge Viterbo de Ferreira 228, 4050-313 Porto, Portugal; 3 Associate Laboratory i4HB—Institute for Health and Bioeconomy, Faculty of Pharmacy, University of Porto, Rua Jorge Viterbo de Ferreira 228, 4050-313 Porto, Portugal; 4 Faculty of Medicine, University of Porto, Alameda Prof. Hernâni Monteiro, 4200-319 Porto, Portugal; 5 CINTESIS—Center for Health Technology and Services Research, Faculty of Medicine, University of Porto, 4050-313 Porto, Portugal; matsoares@med.up.pt; 6 MEDCIDS—Departamento de Medicina da Comunidade Informação e Decisão em Saúde, Faculty of Medicine, University of Porto, 4050-313 Porto, Portugal; 7 Portuguese Red Cross Health School Lisbon, Avenida de Ceuta nº 1, 1300-125 Lisbon, Portugal; 8 Cross I&D, Avenida de Ceuta nº 1, 1300-125 Lisbon, Portugal; 9 Centre for Health Evaluation & Research/Infosaúde, National Association of Pharmacies, 1300-125 Lisbon, Portugal; jose.guerreiro@anf.pt (J.P.G.); ruben.pereira@anf.pt (R.P.); ateixeirarodrigues@med.uminho.pt (A.T.-R.); 10 Life and Health Sciences Research Institute (ICVS), School of Medicine, University of Minho, Campus de Gualtar, 4710-057 Braga, Portugal; 11 ICVS/3Bs PT Government Associate Laboratory, Campus de Gualtar, 4710-057 Braga, Portugal

**Keywords:** drug utilization data, prevalence, diabetes mellitus, antidiabetics

## Abstract

Observational, cross-sectional prevalence studies are costly and time-consuming. The development of indirect methods estimating prevalence used to obtain faster, less-expensive, and more robust results would be an advantage for several healthcare applications. This study aimed to use the drug dispensing data from community pharmacies to estimate the prevalence of Type 2 Diabetes mellitus (T2DM) in the Portuguese population. A cross-sectional study was conducted using a database of dispensed medicines with an indication for Diabetes mellitus in 2018 and 2021, stratified by geographic region. The methodology was based on a sequential method of acquiring prevalence estimates obtained through exposure to medicines using the daily doses defined per thousand inhabitants per day and adjusted to the rate of adherence to therapy, prescription patterns, and concomitance of antidiabetic drugs. The estimated overall T2DM prevalence in 2018 was 13.9%, and it was 14.2% for 2021. The results show the increased consumption of antidiabetic drugs, with fixed-dose combination antidiabetics and new antidiabetics being particularly important in 2021. This work allowed for the development of a model to obtain the estimated prevalence of T2DM based on drug consumption, using a simple, fast, and robust method that is in line with the available evidence. However, with the recent expanding indications for new antidiabetics, the inclusion of further data in the model needs to be studied.

## 1. Introduction

The knowledge of an accurate estimate of the prevalence of a disease or clinical condition has considerable clinical relevance, as it defines the a priori probability of a diagnosis, supports clinical decision making, enables an understanding of the burden of diseases, and guides future research. An adequate prevalence estimate is also required for health technology assessment (HTA) processes, like medicines, medical devices, therapeutic procedures, or health-related interventions, and to provide information to improve clinical practices or health policymaking [1]. The knowledge of the prevalence of a condition in the HTA context is a central parameter, as its magnitude defines the importance of the medicine/technology to be evaluated in societal, political, economic, and clinical terms [2]. The regulatory dynamics associated with these processes would benefit significantly from the regular and dynamic updating of the prevalence value.

This continuous updating of the prevalence estimation through classical observational studies on the primary data is not feasible as it becomes costly, challenging to implement, and time-consuming [3,4,5,6]. Developing accurate, rapid, and validated methods would add value for clinical and regulatory purposes.

While primary data collection may be onerous and expensive, secondary databases are more easily accessible data sources if they are standardized and informatically compatible between healthcare institutions [7,8]. Drug utilization data can be collected along the drug chain (acquisition, storage, distribution, prescribing, treatment compliance, and treatment outcome) from databases at the regional, national, or international levels. The manufacturers, suppliers, regulatory agencies, inpatient and outpatient health institutions (for prescribing data), and pharmacies (for dispensing data) are all data sources that are commonly used for drug utilization research [9]. Drug consumption data have the advantage of being readily available information. However, as this surrogate information may not directly relate to diagnosis, a methodological challenge emerges [10,11]. The research on drug utilization data is essential to improve the methods’ robustness.

In Portugal, ambulatory prescriptions, whether they come from the NHS or private institutions, are reimbursed in the same way. The pharmacies collect all the dispensing records, and the public reimbursement is then made by the Central Administration of the Health System (ACSS). Additional reimbursements made by private insurance companies can also be applicable. As this information is not always readily or freely available, the resort to pharmacy databases is considered as a simpler option.

For the execution and validation of these methods, it is vital to find a clinical condition with particular features, namely, a high prevalence in the population, chronic in nature, a solid pharmacological relationship with the diagnosed and treated population, and one for which we have excellent clinical and epidemiological knowledge to test the model. We have selected Diabetes mellitus (DM), as this disease complies with these criteria.

As a long-known chronic disease, in 2019, the World Health Organization (WHO) presented an updated classification of DM, excluding the subtypes of Type 1 Diabetes mellitus (T1DM) and Type 2 Diabetes mellitus (T2DM), and adding new types of DM (hybrid forms and unclassified DM) [12]. However, T2DM is the most common form, accounting for 90% to 95% of the total diabetes disease cases [12,13]. As the onset of T2DM is challenging to identify due to its asymptomatic characteristics, it is estimated that one-third to one-half of the patients are undiagnosed [13].

The pharmacological therapy for DM comprises the administration of non-insulin antidiabetic drugs (NADs)—therapeutic subgroups identified by their respective Anatomical Therapeutic Chemical (ATC) classification [14]: biguanides, sulfonylureas, alpha-glucosidase inhibitors, thiazolidinediones, dipeptidyl peptidase 4 (DPP-4) inhibitors, glucagon-like peptide-1 (GLP-1) analogues, sodium-glucose co-transporter 2 (SGLT2); and insulins used for outpatient administration.

The clinical guidelines for T2DM management have been established and frequently updated by several expert medical associations, such as the International Diabetes Federation, the American Diabetes Association, the European Association for the Study of Diabetes, public health national agencies, and the Portuguese Society of Diabetology [15,16,17,18]. The pharmacological approach usually starts with metformin and has evolved to include other NADs in a monotherapy or combination therapy. When indicated, insulin can be added to optimize glycemic control and prevent micro- and macrovascular complications in the long term, avoiding hypoglycemic episodes [18,19]. Polytherapy is frequently used when treating DM, resulting in concomitance, which is defined as administering two or more drugs simultaneously for the same condition. The American Diabetes Association and the European Association for the Study of Diabetes treatment guidelines advise up to three NADs plus insulin until HbA1c control [17]. However, some recent studies, mainly from Asia, aim for quadruple NAD therapy [20,21,22]. Ultimately, the decision is clinical and patient-centered, based on the best available clinical evidence.

This study aimed to estimate the prevalence of T2DM in the Portuguese population. Meanwhile, the evolution of antidiabetic drug (AD) consumption in 2018 and 2021 was characterized according to the ATC code and stratified by region. The impact of off-label use, therapeutic adherence, and concomitant AD prescription in the prevalence estimation was also assessed.

This study was carried out on a representative sample of medicine-consuming people in Portugal, enabling comparison with the existing literature data and providing a simple implementation, allowing easy replication. The development of this type of model for estimating prevalence can be applied to other diseases, and the robustness of the estimation can be improved as the inputs of the model are updated.

## 2. Materials and Methods

A nationwide, descriptive study of drug dispensing data was conducted to allow the estimation of T2DM prevalence in the Portuguese population. To assess the robustness of the estimation, the model was used to estimate the prevalence of T2DM in two periods: the full year 2018 and the full year 2021.

### 2.1. Data Source

ADs for outpatient use are dispensed in Portugal upon prescription only. Portuguese data were retrieved from the Health Market Research^®^ Pharmacy Sales Information System, a national database of around 2400 pharmacies with drug dispensing data (including sales by medicine ATC code, pharmaceutical form, number of units, and dosage) at the regional level for ambulatory care. This database includes data from more than 82% of the Portuguese community pharmacies.

Data collection was based on the ATC classification system, as recommended by the WHO [14].

The data collected covered ATC classes A10A, insulins and analogues, and A10B (blood glucose-lowering drugs, excluding insulins), which were commercialized in Portugal in 2018 and 2021.

### 2.2. Population

The quantification of the study population was retrieved from demographic data for 2018 and 2021 (Table 1), including the total national population by the residence district, from the National Official Statistics Agency.

The WHO recommends the unit of drug measurement, Defined Daily Dose (DDD), for drug utilization monitoring and research [24]. According to their definition, the DDD is the “assumed average daily dose of a drug when used in its main therapeutic indication in adults” [14]. It is defined from the existing experience with the drug recommendations in the literature and from its manufacturers. As measured by the DDD, drug consumption is usually expressed as DDD per 1000 inhabitants per day (DID). The DID calculation allows us to roughly determine the proportion of the population receiving, per day, the standard treatment of a given drug.

The population’s exposure to AD was then assumed to correspond to the population’s consumption and is expressed as DID.

Two outcomes were estimated:

1. The total DDD consumed per year was calculated as follows:

Total DDD consumed = No. of units consumed in one-year × dosage/DDD(1)

The DDD for each drug was based on the ATC Index 2022, regardless of the year the consumption refers to. The drugs were classified according to the ATC Index 2022, level 5—active substance.

2. DID: This indicates the number of people per 1000 who receive antidiabetic drugs daily and was calculated using the following equation.

DID = Total DDD consumed × 1000/(No. inhabitants × 365 days)(2)

### 2.3. Literature Data

The national and international literature data considered most relevant and chosen for comparing the results are compiled in Table 2, along with the bibliographical references.

### 2.4. Prevalence Estimation Model

The estimation of the prevalence of T2DM was performed by applying the formula proposed by Sartor and Walckiers [30]:

(3) $$\hat{p}=\frac{\sum_{i=1}^{k}\left(\frac{v_{i}}{c_{i}}\right)}{N}$$

where:

$\hat{p}$—the estimated prevalence;

k—the classes of drugs;

$v_{i}$—the total amount of NAD sold in Portugal for the years 2018 and 2021;

$c_{i}$—the technical unit of drug consumption (DID);

N—the estimated resident population in Portugal for 2018 and 2021.

For the application of this methodology, the authors assumed the following assumptions:

- The treatment of the pathology is mainly pharmacological;
- The drugs used are specific for the pathology considered;
- The administration of the drugs is not seasonal.

The prevalence estimation model was processed using Microsoft Office Excel 2007 software and developed in 5 steps, as follows:

Step (1) The number of patients undergoing treatment for DM based on DID estimations.

Exposure to AD is expressed in DID, which reflects the number of DDDs dispensed to 1000 inhabitants per day. As the DDD is the assumed average daily dose of a drug when used in its main therapeutic indication in adults, it is a rough estimation of the number of patients undergoing treatment with ADs.

The calculations considered the ATC classification—level 4—chemical subgroup.

Step (2) The adjustment to treatment adherence.

Treatment adherence was assumed to be 60% for all the NADs (crude value) [26,27]. It was considered 100% for the insulin AD.

Step (3) The adjustment to NADs in monotherapy or polytherapy.

The concomitance of different antidiabetic pharmacological regimens impacts the prevalence estimation, i.e., two packages in a month may be used to treat one patient or two patients, according to the therapeutic regimen.

Concomitance in NAD therapy was estimated according to Sartor and Walckiers, 1995 [30], by using the weighting factor (*w*), which was achieved as follows:

(4) $$w=\frac{1}{\left[1+\pi_{2}+\left(2\pi_{3}\right)+\left(3\pi_{4}\right)\right]}$$

where $\pi_{2}$, $\pi_{3}$, and $\pi_{4}$ represent the probability of patients taking two, three, or four drugs simultaneously, respectively.

We applied a *w* value of 0.608 that corrects for the proportion of patients taking an association of two, three, or four classes of NAD following an oral hypoglycemic drug use study [28].

Step (4) The exclusion of insulin AD users.

Insulin-only users were excluded. Regarding the T2DM patients, it was considered that 7.5% of them were insulin AD users [29].

Step (5) The prevalence estimation of T2DM patients under pharmacological treatment

Prevalence was obtained as a ratio of the number of patients undergoing treatment for T2DM estimated in the previous steps per Portuguese patient [23].

Step (6) The prevalence estimation of T2DM.

To obtain an overall estimation of the prevalence of T2DM in Portugal, the percentages of non-diagnosed T2DM were considered: 44% for 2018 [25] and 35.7% for 2021 [13] of non-diagnosed patients.

### 2.5. Sensitivity Analysis

Sensitivity analysis was performed by varying the crucial parameters used in base case analysis. Each of these assumptions was changed, while all the other variables were held constant.

We assessed the effect on T2DM estimated prevalence of changing the following parameters for the extreme values found in the literature:

- Medication adherence: 50% and 92% [31,32,33];
- T2DM insulin users: 6% and 15.8% [29,34];
- The concomitant factor: *w* = 0.596 [35];
- Non-diagnosed T2DM patients: 9.8% and 50% [13].

## 3. Results

### 3.1. Characterization of AD Consumption in Portugal

**Figure 1:**
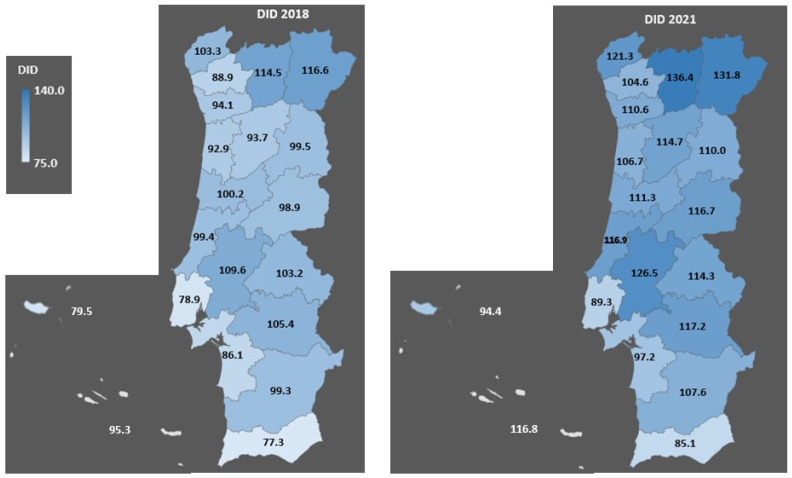
Consumption of antidiabetic drugs expressed by Defined Daily Dose per 1000 inhabitants per day by region in 2018 and 2021.

Figure 1 shows the AD consumption rates measured in DID in Portugal and its regions in 2018 and 2021.

The consumption of ADs in Portugal in 2018 and 2021 increased from 91 DID to 104.9 DID, presenting an increase in all of the regions. The region of the lowest consumption rate in 2018 was Lisbon, and in 2021, it was Faro, while the highest one occurred in Bragança in both these periods.

The insulin consumption rate increased in 2018 and 2021 from 15.5 DID to 16.0 DID (% change of +2.6%), while the NAD consumption rate experienced an increase from 75.4 DID to 88.9 DID (% change of +17.9%), respectively (Table 3).

The regions with the highest NAD consumption rates in 2018 were Vila Real and Bragança, and similar ranked positions were found in 2021. Lisbon and Faro were the regions with the lowest NAD consumption rates in 2018 and 2021.

In 2018, insulins represented 17.0% of the total ADs consumed, and in 2021, this decreased to 15.3%. NAD use increased from 82.9% to 84.8% from 2018 to 2021. Among the NADs, sulphonylureas showed a more considerable decrease in consumption, and the oral fixed-dose combinations, DPP-4 inhibitors, GLP-1 analogues, and SGLT2 inhibitors, presented a growth in their consumption.

Table 3 shows that there was a change in the pattern of NAD consumption between 2018 and 2021. Biguanides and oral fixed-dose combinations are the most commonly consumed subgroups in the two years analyzed. However, the consumption rate of sulfonylureas is decreasing, and the trend is increasing for the newest NADs: DPP-4 inhibitors, GLP-1 analogues, and SGLT2 inhibitors.

### 3.2. Estimated Prevalence of Diabetes in Portugal

The number of patients undergoing treatment and the AD consumption rate for T2DM increased for every region and national area between 2018 and 2021. The prevalence of treated T2DM has also increased, but not in the case of overall prevalence when considering the non-diagnosed patients. The Coimbra, Lisboa, and Setubal regions experienced no variation in overall prevalence. For the regions of Beja, Évora, Faro, and Guarda e Portalegre, this value has even reduced despite the growth at the national level (Table 4).

Using an indirect method to estimate the prevalence based on AD consumption, that of T2DM was 7.8% in 2018 and 9.1% in 2021 for the patients undergoing pharmacological treatment.

The overall estimated prevalences considering the assumed proportion of non-diagnosed patients described in the Methods were 13.9% and 14.2% in 2018 and 2021, respectively.

### 3.3. Sensitivity Analysis

Table 5 shows the prevalence estimates obtained by changing some parameters through sensitivity analysis.

## 4. Discussion

This study obtained estimated overall prevalences of T2DM of 13.9% and 14.2% (2018 vs. 2021) and 7.8% and 9.1% (2018 vs. 2021) for the patients undergoing treatment, respectively. An easy-to-implement-and-to-update method was used to estimate the prevalence of clinical conditions without resorting to the primary data or databases that are difficult to obtain and complex to compute.

### 4.1. Characterization of AD Consumption

The AD consumption rate increased between 2018 and 2021, with overall values of 91 DID and 104.9 DID, respectively. The NADs showed more pronounced growth than the insulins, rising from 75.4 DID in 2018 to 88.9 DID in 2021, and the insulins maintained their value at 15.5–16.0 DID. The pattern of NAD consumption changed between 2018 and 2021, with a decrease in sulphonylureas and a growth in combination ADs and DPP-4 inhibitors, GLP-1 analogues, and SGLT2 inhibitors. In 2018, the NADs that showed the most consumed ones were biguanides (24.2 DID), followed by dose-fixed combinations (22.4 DID).

The 2019 National Diabetes Observatory Annual Report reported an overall AD consumption of 68.1 DID for 2017. The Organisation for Economic Co-operation and Development (OECD) data for pharmaceutical consumption published DID rates of 70.2 (2018), 74.0 (2019), and 76.6 (2020) [36]. These differences may be explained by evaluating different metrics (e.g., the calculation of DID of fixed-dose combinations) and databases (population covered).

Although lifestyle can be a cause of T2DM, and its control may have a therapeutic effect [37,38,39], there is still a lack of quality evidence for the most effective lifestyle programs [40,41] and the pharmacological approach required. No results were found about an estimate for the patients treated only with a lifestyle approach, especially in real-world clinical practice.

Regarding the growth in consumption of the most recent AD agents, the trend is also increasing across Europe, as shown by a study of 11 European countries [42]. Some reasons can explain the increase in AD consumption, such as the increased prevalence of DM, increased longevity, increased early diagnosis and consequent increase in treated patients, the possible treatment of situations classified as pre-diabetes, more aggressive clinical approaches with higher doses, changes in prescribing patterns in line with new guidelines, and the emergence of fixed-dose NAD combinations following the growing scientific evidence. The increased prevalence of obesity in childhood and adolescence also changes the epidemiological picture of T2DM, with its onset at increasingly earlier ages [43].

It should be pointed out that in recent years, new ADs, such as GLP-1 agonists and SGLT2 inhibitors, expanded their indications beyond T2DM. This is the case of semaglutide for obesity [44,45], tirzepatide for obesity and its co-morbidities [46], and dapaglifozin and empaglifozin for heart failure and chronic kidney disease [44]. However, most of them were approved in Europe after 2021, and for this reason, were not considered as such in this study. Nevertheless, dapaglifozin was approved by the EMA for heart failure in November 2020 and chronic kidney disease in the middle of 2021 [44]. In Portugal, the decision to reimburse was authorized in 2022 [47]. These facts make their use other than for T2DM considered residual at the time of data collection.

The geographical distribution of AD consumption remains constant across the Portugal regions when comparing 2018 with 2021. The increasing trend was seen across the regions and with similar distributions.

### 4.2. Prevalence Estimation

This study found an overall and treated estimated prevalence of T2DM of 13.9% and 7.8% for 2018 and 14.2% and 9.1% for 2021, respectively.

Between 2006 and 2007, the Epidemiological Study on the Prevalence of Metabolic Syndrome in the Portuguese Population (VALSIM) was carried out [34]. This cross-sectional study used a representative sample of adults living in mainland Portugal and the islands followed up in primary healthcare facilities, and 16,856 individuals were evaluated, with the registration of their previous diagnosis and the control of fasting glycemia and HbA1c, out of several other parameters. Among the characterization of cardiovascular risks, the prevalence, treatment, and control of DM were evaluated. The prevalence of DM in the population using primary healthcare services, adjusted for sex and age, was 14.9%, increasing progressively with age and being higher in men (men: 16.8%; women: 13.2%). The limitations of this study are related to the sampling method, which was conducted by convenience in primary healthcare facilities, not including the individuals who do not seek public healthcare and may have undiagnosed DM.

The data from the General Practice Sentinel Network between 1992 and 2015 were used to describe the evolution of DM incidence and to estimate the future incidence of DM through Poisson regression models until 2024. The average increase in DM incidence rate was 4.29% [confidence interval (CI) 95%: 3.80–4.80], and the study revealed an increase in incidence projections similar to the observed data. The reasons were attributable to the population ageing and the higher-risk groups [48].

The results from the First National Health Examination Survey (INSEF 2015) showed an overall prevalence of diabetes of 9.9% (CI95%: 8.4; 11.5), again with a higher value in the males when compared with that of the females (12.1% vs. 7.8%). This cross-sectional study was performed on a sample of 4911 adults from Portuguese primary healthcare centers at the national level through interviews, physical examination, and blood analysis (for HbA1c, among others) [49].

The Portuguese National Diabetes Observatory publishes an annual report to generate and disseminate reliable and scientifically credible information on diabetes in Portugal using essentially registered and collected information on diabetes in the Portuguese NHS as a data source. This is based on the first large epidemiological study on DM carried out in the country, the PREVDIAB, with in-person data collection conducted in 2008 on a sample of 5167 individuals. It has been updated with population data from the national censuses [50]. The latest annual report of the national diabetes observatory published in 2019 estimated a DM prevalence of 13.6%. However, its main limitation is the voluntary participation of NHS patients and the fact that private healthcare entities were not considered.

The International Diabetes Federation performs frequent estimates of diabetes prevalence for several countries based on the highest-quality data available during analysis. These estimates depend on the type of data sources, ranging from peer-reviewed publications to national survey data, or data from regulatory bodies. The data quality highly depends on the diagnostic method chosen for DM, the sample size and representativeness, the study’s date, the type of study, and the publication type. Predicted future estimates are based only on the projected changes in age, sex, and rural/urban place of residence, as defined by the United Nations [51]. The International Diabetes Federation prevalence estimates for Portugal for 2018 and 2021 were 14.2% and 13%, respectively.

Compared to the European data, the prevalence of T2DM is much higher in Portugal (Table 2). It is hypothesized that this may be due to the adoption of less-healthy lifestyles inherent in a consumer society, but mainly to the countries’ demographics. Portugal is a country with the fourth highest proportion of elderly people in Europe, with an aging index (the ratio between the elderly population (over 65) and the young population (under 14)) of 169.4 in 2019 and 181.3 in 2021 [23]. Consequently, the overload on primary healthcare may be becoming less effective in preventing the disease, together with a lack of health literacy among this population segment.

This study reached a result that falls within the estimates considered for comparison. However, this is not precisely the same trend since the International Diabetes Federation predicts a decrease in prevalence in Portugal in 2021, contrary to the results obtained, which probably reflect more complex prescription patterns.

### 4.3. Prevalence Estimation Model: Adherence to Medication in DM

Adherence to therapy is an important parameter reflected in drug consumption and the consequent effectiveness of the prescribed therapy. Non-adherence can be defined as the failure to follow the medical prescription, usually classified as primary (failure to fill a new prescription) versus secondary non-adherence (failure on medication use upon filling a prescription) [52].

It is challenging to assess adherence in DM, and several studies diverge in terms and metrics to achieve a consensus. Pasina et al. obtained a non-adherence rate reported (n = 100) in 55.1% of the patients at the first follow-up and 69.6% 3 months from hospital discharge in elderly patients receiving polypharmacy, confirming that age is a predictor of increased non-adherence [53,54]. A survey performed in 2016 in Slovakia (n = 117) found adherence rates to pharmacological treatment of up to 83% in females and 79% in males and over 70% for all the age categories up to 75 years old [33]. In Italy, a study conducted in 2017 obtained rates of non-adherence ranging from 8% to 13% for AD [32]. According to the WHO report of 2003, the average adherence to long-term therapy for chronic diseases in developed countries is approximately 50%, and in developing countries, lower adherence rates were encountered [26,31]. Some systematic reviews approached the adherence of NAD and performed interesting comparisons between them, but none provided a crude adherence estimate as a reference [55,56,57]. A recent cross-sectional study on adherence in chronic diseases performed in Spain found that the proportion of patients adherent to their treatment was 55.5% [27].

Regarding the study of primary adhesion, Raebel et al., in 2012, performed a retrospective, observational cohort study to identify primary non-adherence for hypertension, diabetes, and hyperlipidemia patients. The proportion of primary non-adherent patients varied by therapeutic class, and a value of 13% was found to be the primary non-adherence rate for antidiabetic drugs [58].

The studies on adherence to ADs are limited, and none applied to the Portuguese population were found. In this study, the knowledge of the primary adherence reflected in dispensing the prescribed drugs is of paramount interest. Considering the range of estimations published in the literature, a global treatment adherence rate (either primary or secondary) of 60% was considered as the base case scenario, taking into consideration the closest scenario found in a European country for chronic diseases [27].

### 4.4. Prevalence Estimation Model: Concomitant Use of AD Drugs

For the application of the model, it was also necessary to adjust for the concomitant use of ADs, according to the most recent guidelines for DM treatment management, which sometimes may reach two, three, or four medications.

Some studies about prescribing patterns have been performed in recent years, but only a few reflect the usage of new NADs. Heintjes et al. conducted an observational study analyzing the Electronic Health Records of antidiabetic drug prescription/dispensing in four European countries (Netherlands, Spain, Italy, and the United Kingdom) from 2007 to 2012 (n = 295,923). The prescription patterns were categorized as first-line: oral monotherapy (metformin, a sulphonylurea, a thiazolidinedione, DPP4 inhibitors, other oral antidiabetic drugs, a GLP1, or insulin); second line: dual oral therapy (combined metformin + sulphonylurea, any oral combination treatment including a thiazolidinedione, and any oral combination treatment, including a DPP4 inhibitor); third line: triple oral therapy (oral/insulin or oral/GLP-1 combination therapy); fourth line: insulin only, a GLP-1 only, or a combination of insulin + GLP-1. Combining all the countries, 47% initiated a first-line treatment, 28% initiated a second-line treatment, 19% initiated a third-line treatment, and 7% progressed to a fourth-line treatment [35].

In Italy, a regional observational study was performed in 2016 (n = 14,679) of new users of ADs, considering only first-line treatments (monotherapy, fixed-dose combination and dual therapy). The results showed dispensing rates of 86.9% for the monotherapy, 9.7% for the fixed-dose combination therapy, and 3.4% for the dual therapy. However, this study did not account for therapy intensification, that is, second-, third-, or fourth-line treatments [59]. Another study conducted in Spain in 2013–2014 (n = 65,167) obtained similar results [60]. Some other researchers are dedicated to assessing the same subject, though not achieving a general prescription pattern for concomitance [60,61].

The prevalence of insulin monotherapy in T2DM in Europe is unknown, but a study from 2018 estimated that the T2DM insulin users’ values in the United Kingdom, Sweden, and Denmark were 12.5%, 11.7%, and 15.8%, respectively. An estimated 7.5% of T2DM insulin users in Europe were reported [29]. In a study from the United States of America conducted in 2014, 12% of patients with T2DM used insulin monotherapy and 14% used insulin combined with an NAD [62]. Based on a study from the United Kingdom [63], in 2010, 37% of T2DM patients were treated with insulin. From the VALSIM study, of the 3215 people with diabetes, 90.2% were on a targeted treatment, meaning 9.8% of the subjects were untreated. Among the treated patients, 89% were on oral antidiabetics alone, 6% were on insulin therapy, and 5% were on simultaneous oral antidiabetic and insulin therapy [34]. The scenario has changed with the market authorization of new hypoglycemic agents [64].

In this study, the weight factor (*w*) for medicine associations was used for the calculation methodology developed by Sartor et al., 1995 [30], based on a study by Duarte-Ramos et al. [28]. The same methodology has been used by other authors [65]. The weight factor used (*w* = 0.608) was obtained in a national cross-sectional survey from December 2003 until March 2004. Considering the study period, it is easy to notice that the prescribing patterns do not correspond to the current ones, namely polytherapy. The mentioned study by Heintjes et al. [35] obtained updated prescribing patterns, and their results were used to recalculate the weight factor for the associations. The factor obtained (*w* = 0.596) yielded estimated prevalence values for the treated T2DM patients of 7.6% for 2018 and 9.0% for 2021, proving to have a little influence on the final estimation. The weight factor of Duarte-Ramos [28] obtained for the Portuguese population was then considered. It is important to consider the periodic collection of primary data on prescription patterns for longitudinal monitoring. This will allow for information acquisition to calculate the correction factor *w* and adjust the estimates.

A proportion of non-treated DM patients (44%) was considered based on the National Diabetes Observatory Annual Report for the Portuguese population in 2018 [25]. A value of 35.7% was considered for 2021 based on the International Diabetes Federation for Europe [13]. This estimate may not be accurate, considering that it was acquired from in-person surveys.

### 4.5. Sensitivity Analysis

Sensitivity analysis was conducted based on the parameter values found in the literature. The choice of the base case scenario was based on the closest approximation to the Portuguese population, the data origin (official sources and professional associations), and the closeness to the years studied. Subsequently, the other scenarios were developed by varying the parameters between the lowest and highest values found in the literature. The results show that the parameters most affected the DM prevalence were medication adherence and the undiagnosed T2DM patients due to their more significant variation. These parameters became critical in the obtention of an accurate result.

Medication adherence is a parameter that influences AD consumption, and for which there are no updated estimations for the Portuguese population. The existing information shows a considerable variation between the studies. The adherence considered to be 60% for all the NADs and 100% for the insulins indeed does not correspond to real-world since there is some non-adherence in insulins, although this is not significant. On the other hand, adherence among NAD users is not homogeneous, since it is directly linked to adverse effects and costs, which are very different among the ATC subclasses.

### 4.6. Strengths and Limitations

As a limitation, this model can only estimate the prevalence of diagnosed and treated DM due to the data’s origin.

Concerning the medications utilized on a continuous or chronic basis, such as antidiabetics or antihypertensives, the DID is a rough estimation of the number of patients undergoing chronic treatment since it is a technical unit of measurement of the exposure of a given population to a particular drug to perform the aggregation of drug subgroups and geographical comparisons [10,66]. The access to aggregate data requires parameter adjustments with assumptions that are not always precise and appropriate to the study population, influencing their validity. Although the DID is an estimate of the actual use of the drugs, it does not necessarily reflect the prescribed daily dose (which depends on the posology) or the daily dose consumed (which is related to treatment compliance). For the same reason, the outcome cannot be stratified by sex or age as clinically valuable information.

Therapeutic adherence, which reflects compliance to a prescription, is reflected in the data on drug dispensing, the implementation of the correct treatment, and the persistence of the same treatment that impacts the prevalence estimation, and high-quality adherence studies should be considered [56,67]. Although they are not very suitable for identifying the direct non-adherence cases, drug dispensing records are more accessible than prescription and clinical records for the indirect calculation of adherence and its indicators.

The aggregate drug consumption data do not precisely reflect the prescription clinical practice, which implies predicting dosing patterns and the concomitant use of drugs [21,68]. The prescribing patterns have changed over the years, and the concomitance factor used will not be updated for the emergence of new NADs and combinations, with the increase in polytherapy and the trend towards the introduction of increasingly earlier insulin use for the control of hyperglycemia and the prevention of future complications.

The off-label use of medicines distorts the drug consumption data and their approved indications, and in most cases, their alternative application is unknown. The off-label use of a drug is defined as the prescription of a drug outside the scope of the therapeutic indications approved in the respective marketing authorization [69]. This implies that it has yet to be assessed, or at least validated, by the health authorities and, therefore, lacks the same scientific evidence as the treatments approved for specific indications. Consequently, the data about the off-label use of medicines are scarce, as it is often associated with a practice with no legal framework [69]. The off-label use of ADs is not well documented, but some studies suggested the use of NADs for T1DM [70,71,72,73,74,75], obesity [70,76], polycystic ovarian syndrome [77,78,79], dermatological diseases [80,81], and as a senolytic [82,83,84]. Recently, the European Medicines Agency approved the SGLT inhibitors dapagliflozin and sotagliflozin as adjuvant treatments to insulin for T1DM in adults [85], and insulins were already recommended in T2DM in the previous guidelines. In this study, the off-label use of NADs was not considered since no data metrics were found.

The recently expanded indications for GLP-1 agonists and SGLT2 inhibitors were not reflected in this study, as it refers to data from up to 2021. However, the implementation of the developed model at the current time requires the inclusion of prescription data for the additional indications approved to date.

Although comprehensive research is conducted in robust sensitivity analysis, the estimates used for several parameters were based on the available literature, which is only sometimes updated, is related to other populations or does not use robust methods, biasing the final estimated result.

The database used does not include inpatient consumption; however, it includes other health subsystems that need to be identified in the NHS databases.

The indirect method of estimating the prevalence of drug utilization data has been tested in several chronic diseases [86,87,88,89], such as ocular hypertension [90], inflammatory bowel disease [65], thyroid disorders [91], and DM [28,92,93]. Compared with the other studies using prescription data or data not covering the entire population, the dispensing information obtained from a sample representing 82% of the pharmacies in Portugal with a homogeneous geographical distribution is a strength of the study consolidating the results presented.

This method allowed us to estimate DM prevalence simply. This will benefit from evolving drug utilization research, especially studies on primary and secondary adherence, prescribing patterns, and the off-label use of AD, to refine the assumed parameters.

## 5. Conclusions

An estimate of T2DM prevalence was achieved from aggregate AD drug consumption data, with similar results to those obtained by the standard methodologies. Nevertheless, the developed method operates on many assumptions and can be improved by more precise estimates for the assumed parameters to substitute or complement the traditional methods for estimating the prevalence of clinical conditions.

The developed method based on information collected from the pharmacy network widely distributed throughout Portugal has the advantage of accessing a large and representative population sample size, can be repeated periodically, and can be regionally stratified, identifying and exploring the differences for this purpose.

This study contributes to the development of pharmacoepidemiology and drug utilization data. Less-complex methods, even if not as accurate than classical methods, allow for the fast updating of health policies, clinical guidelines, or pharmacoeconomic studies.

## Figures and Tables

**Table 1:** Portugal’s population per district [23].

Region	2018	2021
Aveiro	695,702	700,957
Beja	141,178	144,465
Braga	828,650	846,418
Bragança	124,571	122,826
Castelo Branco	179,038	177,995
Coimbra	405,267	408,609
Évora	152,865	152,511
Faro	438,864	467,475
Guarda	144,354	142,998
Leiria	454,592	458,672
Lisboa	2,271,772	2,275,846
Portalegre	105,479	104,930
Porto	1,778,146	1,785,627
Santarém	429,719	425,025
Setubal	852,328	874,926
Viana do Castelo	230,954	231,293
Vila Real	191,894	185,705
Viseu	354,453	351,315
Madeira	253,945	250,769
Açores	242,846	236,440
Total	10,276,617	10,344,802

**Table 2:** Literature data.

		2018	2021	References
Adult diabetes prevalence estimates (20–79 years old)	Global	9.3%	10.5%	[13]
	Europe	8.9%	9.2%	[13]
	Portugal	14.2%	13%	[13]
	Portugal	13.6%		[25]
Non-diagnosed DM	Global	50.1%	44.7%	[13]
	Europe	40.7%	35.7%	[13]
	Portugal/TID	475.2	433.3	[13]
	Portugal	44%		[25]
T2DM treatment adherence		60%	60%	[26,27]
T2DM concomitance factor		0.608	0.608	[28]
T2DM insulin users		7.5%	7.5%	[29]

DM: Diabetes mellitus; T2DM: Type 2 Diabetes mellitus; TID: thousand inhabitants per day.

**Table 3:** Antidiabetic consumption in DID in 2018 and 2021.

		DID (%) ^1^		
ATC Code	Description of the ATC Code	2018	2021	Variation ^2^ (2018–2021, %)
A10AB	Fast-acting insulins	2.8 (3.1%)	3.3 (3.1%)	16.8
A10AC	Intermediate-acting insulins	1.8 (2.0%)	1.3 (1.2%)	−26.8
A10AD	Combined-acting insulins	4.0 (4.3%)	3.3 (3.1%)	−16.8
A10AE	Long-acting insulins	7.0 (7.7%)	8.1 (7.7%)	15.3
A10A	Insulins	15.5 (17.0%)	16.0 (15.2%)	2.6
A10BA	Biguanides	24.2 26.6%)	24.5 (23.4%)	1.4
A10BB	Sulphonylureas	14.5 (15.9%)	11.5 (11.0%)	−20.5
A10BD	Oral fixed-dose combinations	22.4 (24.6%)	28.3 (27.0%)	26.5
A10BF	α-glucosidase inhibitors	0.7 (0.8%)	0.4 (0.4%)	−45.7
A10BG	Glitazones	0.4 (0.4%)	0.4 (0.4%)	−11.7
A10BH	DPP-4 inhibitors	7.0 (7.7%)	7.6 (7.2%)	8.9
A10BJ	GLP-1 analogues	1.6 (1.8%)	4.9 (4.7%)	196.8
A10BK	SGLT2 inhibitors	4.4 (4.8%)	11.2 (10.7%)	153.0
A10BX	Other non-insulin antidiabetics	0.2 (0.2%)	0.1 (0.1%)	−40.1
A10 B	Non-insulin antidiabetics	75.4 (82.9%)	88.9 (84.8%)	17.9
Total A10	Antidiabetics	91.0	104.9	15.3

^1^ Shared DIDs of each drug in overall AD consumption; ^2^ absolute variation in DID from 2018 to 2021. The raw data was used in the computations. Results from calculations using the variables in Table 3 may vary slightly.

**Table 4:** Estimated prevalence of Type 2 Diabetes mellitus from antidiabetic consumption data.

	N. Patients in Treatment for DM ^1^		N. Patients under Treatment for T2DM ^2^		Prevalence of Treated T2DM Patients (%) ^3^		Overall Prevalence of T2DM (%) ^4^	
Region	2018	2021	2018	2021	2018	2021	2018	2021
Aveiro	65,315	75,645	54,322	64,443	7.8	9.2	13.9	14.3
Beja	14,173	15,723	12,159	13,542	8.6	9.4	15.4	14.6
Braga	74,463	89,563	63,971	78,413	7.7	9.3	13.8	14.4
Bragança	14,678	16,368	12,379	14,070	9.9	11.5	17.7	17.8
Castelo Branco	17,882	20,991	14,429	17,555	8.1	9.9	14.4	15.3
Coimbra	41,017	45,944	32,594	37,924	8.0	9.3	14.4	14.4
Évora	16,286	18,073	14,000	15,766	9.2	10.3	16.4	16.1
Faro	34,275	40,214	28,303	34,120	6.4	7.3	11.5	11.4
Guarda	14,510	15,904	12,101	13,615	8.4	9.5	15.0	14.8
Leiria	45,654	54,204	38,138	46,200	8.4	10.1	15.0	15.7
Lisboa	181,282	205,529	153,755	177,216	6.8	7.8	12.1	12.1
Portalegre	11,007	12,126	9340	10,482	8.9	10.0	15.8	15.5
Porto	169,066	199,690	143,733	173,001	8.1	9.7	14.4	15.1
Santarém	47,601	54,357	40,418	46,830	9.4	11.0	16.8	17.1
Setúbal	74,195	85,955	62,095	73,303	7.3	8.4	13.0	13.0
Viana do Castelo	24,114	28,366	20,824	24,827	9.0	10.7	16.1	16.7
Vila Real	22,214	25,621	19,369	22,760	10.1	12.3	18.0	19.1
Viseu	33,572	40,745	28,179	35,033	7.9	10.0	14.2	15.5
Madeira	20,411	23,940	17,904	21,493	7.1	8.6	12.6	13.3
Açores	23,377	27,908	19,295	23,595	7.9	10.0	14.2	15.5
Total	945,090	1,096,864	797,307	944,187	7.8	9.1	13.9	14.2

DM—Diabetes mellitus; T2DM—Type 2 Diabetes mellitus. ^1^ Based on DID estimation and adjustment to adherence and therapeutic concomitance. ^2^ Exclusion of people with T2DM that are insulin users. ^3^ Estimated prevalence of T2DM-treated patients based on AD consumption. ^4^ Overall estimated prevalence of T2DM considering non-diagnosed patients.

**Table 5:** Sensibility analysis of estimated prevalence of Type 2 Diabetes mellitus.

		Prevalence of Treated T2DM Patients (%)		Overall Prevalence of T2DM (%)	
Parameters	Scenario	2018	2021	2018	2021
Base estimated prevalence ^1^		7.8	9.1	13.9	14.2
Medication adherence	50%	9.3	10.9		
	92%	5.1	6.0		
T2DM insulin users	6.0%	7.9	9.3		
	15.8%	7.7	9.1		
Concomitance factor (*w*)	0.596	7.6	9.0		
Non-diagnosed T2DM patients	9.8%			8.6	10.1
	50%			15.5	18.3

T2DM—Type 2 Diabetes mellitus. ^1^ Base case scenario parameters: medication adherence 60%, and T2DM insulin users 7.5%; *w* factor 0.608; non-diagnosed patients 44% (2018) and 35.7% (2021).

## Data Availability

The data supporting this study’s findings are available from the authors, but restrictions apply to the availability of these data, which were used under license from the National Association of Pharmacies for the current study and are not publicly available. The data are, however, available from the authors upon reasonable request and with permission from the National Association of Pharmacies.

## References

[1] Wulsin L., Dougherty A., California State Library, California Research Bureau (2008). A Briefing on Health Technology Assessment.

[2] Commission E. Health Technology Assessment. https://health.ec.europa.eu/health-technology-assessment_en.

[3] Johnson L.L., Gallin J.I., Ognibene F.P., Johnson L.L. (2018). Chapter 17—Design of Observational Studies. Principles and Practice of Clinical Research.

[4] Wickham R.J. (2019). Secondary Analysis Research. J. Adv. Pract. Oncol..

[5] Pederson L., Vingilis E., Wickens C., Koval J., Re M. (2020). Use of secondary data analyses in research: Pros and Cons. J. Addict. Med. Ther. Sci..

[6] Silman A.J., Macfarlane G.J., Macfarlane T., Silman A.J., Macfarlane G.J., Macfarlane T., Silman A.J., Macfarlane G.J., Macfarlane T. (2018). 90Use of secondary data. Epidemiological Studies: A Practical Guide.

[7] Wettermark B., Zoëga H., Furu K., Korhonen M., Hallas J., Nørgaard M., Almarsdottir A., Andersen M., Andersson Sundell K., Bergman U. (2013). The Nordic prescription databases as a resource for pharmacoepidemiological research—A literature review. Pharmacoepidemiol. Drug Saf..

[8] Hallas J., Hellfritzsch M., Rix M., Olesen M., Reilev M., Pottegård A. (2017). Odense Pharmacoepidemiological Database: A Review of Use and Content. Basic Clin. Pharmacol. Toxicol..

[9] Wettermark B., Elseviers M., Almarsdottir A.B., Andersen M., Benko R., Bennie M., Eriksson I., Godman B., Krska J., Poluzzi E. (2016). Introduction to Drug Utilization Research. Drug Utilization Research: Methods and Applications.

[10] Elseviers M., Wettermark B., Almarsdottir A.B., Andersen M., Benko R., Bennie M., Eriksson I., Godman B., Krska J., Poluzzi E. (2016). Drug Utilization Research: Methods and Applications.

[11] Wettermark B., Vlahović-Palčevski V., Lee D., Bergman U. (2019). Studies of Drug Utilization. Pharmacoepidemiology.

[12] WHO (2019). Classification of Diabetes Mellitus.

[13] IDF (2021). International Diabetes Federation. Diabetes Atlas.

[14] WHO Collaborating Centre for Drug Statistics Methodology Guidelines for ATC Classification and DDD Assignment 2022. https://www.whocc.no/atc_ddd_index_and_guidelines/atc_ddd_index/.

[15] Draznin B., Aroda V.R., Bakris G., Benson G., Brown F.M., Freeman R., Green J., Huang E., Isaacs D., Kahan S. (2022). 9. Pharmacologic Approaches to Glycemic Treatment: Standards of Medical Care in Diabetes-2022. Diabetes Care.

[16] Rui Duarte M.M., Silva Nunes J., Melo P.C., Raposo J.F., Carvalho D., Pelo Grupo de Trabalho para as Recomendações Nacionais da SPD sobre a Terapêutica da Diabetes Tipo 2 (2018). SPD National Recommendations for the Treatment of Hyperglycemia in Type 2 Diabetes—Update Based in the ADA/EASD Joint Position Statement. Rev. Port. Diabetes.

[17] Davies M.J., D’Alessio D.A., Fradkin J., Kernan W.N., Mathieu C., Mingrone G., Rossing P., Tsapas A., Wexler D.J., Buse J.B. (2018). Management of Hyperglycemia in Type 2 Diabetes, 2018. A Consensus Report by the American Diabetes Association (ADA) and the European Association for the Study of Diabetes (EASD). Diabetes Care.

[18] IDF (2012). International Diabetes Federation: Global Guideline for Type 2 Diabetes.

[19] Inzucchi S.E., Bergenstal R.M., Buse J.B., Diamant M., Ferrannini E., Nauck M., Peters A.L., Tsapas A., Wender R., Matthews D.R. (2012). Management of hyperglycemia in type 2 diabetes: A patient-centered approach: Position statement of the American Diabetes Association (ADA) and the European Association for the Study of Diabetes (EASD). Diabetes Care.

[20] Cho Y.K., Lee J., Kim H.S., Park J.-Y., Jung C.H., Lee W.J. (2020). Clinical Efficacy of Quadruple Oral Therapy for Type 2 Diabetes in Real-World Practice: A Retrospective Observational Study. Diabetes Ther..

[21] Moon J.S., Suh S., Kim S.S., Jin H.Y., Kim J.M., Jang M.H., Lee K.A., Lee J.H., Chung S.M., Lyu Y.S. (2021). Efficacy and Safety of Treatment with Quadruple Oral Hypoglycemic Agents in Uncontrolled Type 2 Diabetes Mellitus: A Multi-Center, Retrospective, Observational Study. Diabetes Metab. J..

[22] Ku E.J., Lee D.H., Jeon H.J., Oh T.K. (2021). Long-term effectiveness and safety of quadruple combination therapy with empagliflozin versus dapagliflozin in patients with type 2 diabetes: 3-year prospective observational study. Diabetes Res. Clin. Pract..

[23] INE (Instituto Nacional de Estatística). População residente em Portugal. http://www.ine.pt.

[24] Bachhav S.S., Kshirsagar N.A. (2015). Systematic review of drug utilization studies & the use of the drug classification system in the WHO-SEARO Region. Indian J. Med. Res..

[25] SPD (2019). Relatório Anual do Observatório Nacional da Diabetes.

[26] Sabaté E. (2003). Adherence to Long-Term Therapies: Evidence for Action.

[27] Fernandez-Lazaro C.I., García-González J.M., Adams D.P., Fernandez-Lazaro D., Mielgo-Ayuso J., Caballero-Garcia A., Moreno Racionero F., Córdova A., Miron-Canelo J.A. (2019). Adherence to treatment and related factors among patients with chronic conditions in primary care: A cross-sectional study. BMC Fam. Pract..

[28] Duarte-Ramos F., Cabrita J. (2006). Using a pharmaco-epidemiological approach to estimate diabetes type 2 prevalence in Portugal. Pharmacoepidemiol. Drug Saf..

[29] Basu S., Yudkin J.S., Kehlenbrink S., Davies J.I., Wild S.H., Lipska K.J., Sussman J.B., Beran D. (2019). Estimation of global insulin use for type 2 diabetes, 2018–2030: A microsimulation analysis. Lancet Diabetes Endocrinol..

[30] Sartor F., Walckiers D. (1995). Estimate of disease prevalence using drug consumption data. Am. J. Epidemiol..

[31] Wabe N.T., Angamo M.T., Hussein S. (2011). Medication adherence in diabetes mellitus and self management practices among type-2 diabetics in Ethiopia. N. Am. J. Med. Sci..

[32] Spadea T., Onorati R., Baratta F., Pignata I., Parente M., Pannacci L., Ancona D., Ribecco P., Costa G., Gnavi R. (2021). Monitoring adherence to pharmacological therapy and follow-up examinations among patients with type 2 diabetes in community pharmacies. Results from an experience in Italy. PLoS ONE.

[33] Masaryková L., Tesař T., Lehocká U., Bernáthová K. (2020). Evaluation of adherence to treatment in patients suffering from diabetes mellitus. Ceska Slov. Farm..

[34] Cortez-Dias N., Martins S., Belo A., Fiuza M. (2010). Prevalence, management and control of diabetes mellitus and associated risk factors in primary health care in Portugal. Rev. Port. Cardiol..

[35] Heintjes E.M., Overbeek J.A., Hall G.C., Prieto-Alhambra D., Lapi F., Hammar N., Bezemer I.D. (2017). Factors Associated with Type 2 Diabetes Mellitus Treatment Choice Across Four European Countries. Clin. Ther..

[36] OECD Statistics OECD Health Statistics. http://www.oecd.org/health/health-data.htm.

[37] Pot G.K., Battjes-Fries M.C., Patijn O.N., Zijl N.v.d., Pijl H., Voshol P. (2020). Lifestyle medicine for type 2 diabetes: Practice-based evidence for long-term efficacy of a multicomponent lifestyle intervention (Reverse Diabetes2 Now). BMJ Nutr. Prev. Health.

[38] Panigrahi G., Goodwin S.M., Staffier K.L., Karlsen M. (2023). Remission of Type 2 Diabetes after Treatment with a High-Fiber, Low-Fat, Plant-Predominant Diet Intervention: A Case Series. Am. J. Lifestyle Med..

[39] Zhang Y., Yang Y., Huang Q., Zhang Q., Li M., Wu Y. (2023). The effectiveness of lifestyle interventions for diabetes remission on patients with type 2 diabetes mellitus: A systematic review and meta-analysis. Worldviews Evid. Based Nurs..

[40] Churuangsuk C., Hall J., Reynolds A., Griffin S.J., Combet E., Lean M.E.J. (2022). Diets for weight management in adults with type 2 diabetes: An umbrella review of published meta-analyses and systematic review of trials of diets for diabetes remission. Diabetologia.

[41] Kelly J., Karlsen M., Steinke G. (2020). Type 2 Diabetes Remission and Lifestyle Medicine: A Position Statement From the American College of Lifestyle Medicine. Am. J. Lifestyle Med..

[42] Mardetko N., Nabergoj Makovec U., Locatelli I., Janez A., Kos M. (2021). Uptake of new antidiabetic medicines in 11 European countries. BMC Endocr. Disord..

[43] Kansra A.R., Lakkunarajah S., Jay M.S. (2020). Childhood and Adolescent Obesity: A Review. Front. Pediatr..

[44] Agency E.M. European Medicines Agency. Medicines. https://www.ema.europa.eu/en/medicines.

[45] Wojtara M., Mazumder A., Syeda Y., Mozgała N. (2023). Glucagon-Like Peptide-1 Receptor Agonists for Chronic Weight Management. Adv. Med..

[46] Abbasi J. (2023). FDA Green-Lights Tirzepatide, Marketed as Zepbound, for Chronic Weight Management. JAMA.

[47] DATS (Departamento de Avaliação de Tecnologias da Saúde) Relatório Público de Avaliação: FORXIGA (Dapaglifozina) INFARMED. https://www.infarmed.pt/documents/15786/3368817/Relat%C3%B3rio+de+avalia%C3%A7%C3%A3o+de+financiamento+p%C3%BAblico+de+Forxiga+%28dapagliflozina%29/bb0f8fc4-b164-ad19-66d8-67d1b2d70b88.

[48] de Sousa-Uva M., Antunes L., Nunes B., Rodrigues A.P., Simões J.A., Ribeiro R.T., Boavida J.M., Matias-Dias C. (2016). Trends in diabetes incidence from 1992 to 2015 and projections for 2024: A Portuguese General Practitioner’s Network study. Prim. Care Diabetes.

[49] Barreto M., Kislaya I., Gaio V., Rodrigues A.P., Santos A.J., Namorado S., Antunes L., Gil A.P., Boavida J.M., Ribeiro R.T. (2018). Prevalence, awareness, treatment and control of diabetes in Portugal: Results from the first National Health examination Survey (INSEF 2015). Diabetes Res. Clin. Pract..

[50] Gardete-Correia L., Boavida J.M., Raposo J.F., Mesquita A.C., Fona C., Carvalho R., Massano-Cardoso S. (2010). First diabetes prevalence study in Portugal: PREVADIAB study. Diabet. Med..

[51] Aschner P., Karuranga S., James S., Simmons D., Basit A., Shaw J.E., Wild S.H., Ogurtsova K., Saeedi P. (2021). The International Diabetes Federation’s guide for diabetes epidemiological studies. Diabetes Res. Clin. Pract..

[52] Raebel M.A., Schmittdiel J., Karter A.J., Konieczny J.L., Steiner J.F. (2013). Standardizing terminology and definitions of medication adherence and persistence in research employing electronic databases. Med. Care.

[53] Pasina L., Brucato A.L., Falcone C., Cucchi E., Bresciani A., Sottocorno M., Taddei G.C., Casati M., Franchi C., Djade C.D. (2014). Medication non-adherence among elderly patients newly discharged and receiving polypharmacy. Drugs Aging.

[54] Yap A.F., Thirumoorthy T., Kwan Y.H. (2016). Systematic review of the barriers affecting medication adherence in older adults. Geriatr. Gerontol. Int..

[55] McGovern A., Tippu Z., Hinton W., Munro N., Whyte M., de Lusignan S. (2018). Comparison of medication adherence and persistence in type 2 diabetes: A systematic review and meta-analysis. Diabetes Obes. Metab..

[56] Evans M., Engberg S., Faurby M., Fernandes J., Hudson P., Polonsky W. (2022). Adherence to and persistence with antidiabetic medications and associations with clinical and economic outcomes in people with type 2 diabetes mellitus: A systematic literature review. Diabetes Obes. Metab..

[57] Lee D.S.U., Lee H. (2022). Adherence and persistence rates of major antidiabetic medications: A review. Diabetol. Metab. Syndr..

[58] Raebel M.A., Ellis J.L., Carroll N.M., Bayliss E.A., McGinnis B., Schroeder E.B., Shetterly S., Xu S., Steiner J.F. (2012). Characteristics of patients with primary non-adherence to medications for hypertension, diabetes, and lipid disorders. J. Gen. Intern. Med..

[59] Moreno Juste A., Menditto E., Orlando V., Monetti V.M., Gimeno Miguel A., González Rubio F., Aza-Pascual-Salcedo M.M., Cahir C., Prados Torres A., Riccardi G. (2019). Treatment Patterns of Diabetes in Italy: A Population-Based Study. Front. Pharmacol..

[60] Moreno-Juste A., Poblador-Plou B., Aza-Pascual-Salcedo M., González-Rubio F., Malo S., Librero López J., Pico-Soler V., Giménez Labrador E., Mucherino S., Orlando V. (2020). Initial Therapy, Regimen Change, and Persistence in a Spanish Cohort of Newly Treated Type 2 Diabetes Patients: A Retrospective, Observational Study Using Real-World Data. Int. J. Environ. Res. Public Health.

[61] Ingrasciotta Y., Bertuccio M.P., Crisafulli S., Ientile V., Muscianisi M., L’Abbate L., Pastorello M., Provenzano V., Scorsone A., Scondotto S. (2020). Real World Use of Antidiabetic Drugs in the Years 2011–2017: A Population-Based Study from Southern Italy. Int. J. Environ. Res. Public Health.

[62] Wallia A., Molitch M.E. (2014). Insulin Therapy for Type 2 Diabetes Mellitus. JAMA.

[63] Holden S.E., Gale E.A., Jenkins-Jones S., Currie C.J. (2014). How many people inject insulin? UK estimates from 1991 to 2010. Diabetes Obes. Metab..

[64] Ni X., Zhang L., Feng X., Tang L. (2022). New Hypoglycemic Drugs: Combination Drugs and Targets Discovery. Front. Pharmacol..

[65] Azevedo L.F., Magro F., Portela F., Lago P., Deus J., Cotter J., Cremers I., Vieira A., Peixe P., Caldeira P. (2010). Estimating the prevalence of inflammatory bowel disease in Portugal using a pharmaco-epidemiological approach. Pharmacoepidemiol. Drug Saf..

[66] Rasmussen L., Wettermark B., Steinke D., Pottegård A. (2022). Core concepts in pharmacoepidemiology: Measures of drug utilization based on individual-level drug dispensing data. Pharmacoepidemiol. Drug Saf..

[67] Caetano P.A., Lam J.M., Morgan S.G. (2006). Toward a standard definition and measurement of persistence with drug therapy: Examples from research on statin and antihypertensive utilization. Clin. Ther..

[68] Lin Y.-Y., Weng S.-F., Hsu C.-H., Huang C.-L., Lin Y.-P., Yeh M.-C., Han A.-Y., Hsieh Y.-S. (2022). Effect of metformin monotherapy and dual or triple concomitant therapy with metformin on glycemic control and lipid profile management of patients with type 2 diabetes mellitus. Front. Med..

[69] Rusz C.-M., Ősz B.-E., Jîtcă G., Miklos A., Bătrînu M.-G., Imre S. (2021). Off-Label Medication: From a Simple Concept to Complex Practical Aspects. Int. J. Environ. Res. Public Health.

[70] Tsushima Y., Zhou K., Bena J.F., Kashyap S.R. (2022). Prevalence and Clinical Determinants of Obesity in Adults with Type 1 Diabetes Mellitus: A Single-Center Retrospective Observational Study. Endocr. Pract..

[71] Hughes M.S., Bailey R., Calhoun P., Shah V.N., Lyons S.K., DeSalvo D.J. (2022). Off-label use of sodium glucose co-transporter inhibitors among adults in type 1 diabetes exchange registry. Diabetes Obes. Metab..

[72] Sjöholm Å. (2021). Using adjuvant pharmacotherapy in the treatment of type 1 diabetes. Expert Opin. Pharmacother..

[73] Lane K., Freeby M. (2021). Adjunctive therapies in type 1 diabetes mellitus. Curr. Opin. Endocrinol. Diabetes Obes..

[74] El Hage L., Kashyap S.R., Rao P. (2019). Use of SGLT-2 Inhibitors in Patients with Type 1 Diabetes Mellitus. J. Prim. Care Community Health.

[75] Bayona Cebada A., Nattero-Chávez L., Alonso Díaz S., Escobar-Morreale H.F., Luque-Ramírez M. (2020). Efficacy and Safety of SGLT2 Inhibitors in Type 1 Diabetes After the Introduction of an Off-Label Use Protocol for Clinical Practice. Diabetes Technol. Ther..

[76] Sari C., Seip R.L., Umashanker D. (2021). Case Report: Off Label Utilization of Topiramate and Metformin in Patients with BMI ≥ 50 kg/m^2^ Prior to Bariatric Surgery. Front. Endocrinol..

[77] Ayub M., Gul S., Zehra F. (2019). Report: Metformin potential in predisposing arthralgia, type II cross reactivity secondary to group A streptococcus infection & other comorbidities in treating PCOS. Pak. J. Pharm. Sci..

[78] Nathan N., Sullivan S.D. (2014). The utility of metformin therapy in reproductive-aged women with polycystic ovary syndrome (PCOS). Curr. Pharm. Biotechnol..

[79] Facchinetti F., Orrù B., Grandi G., Unfer V. (2019). Short-term effects of metformin and myo-inositol in women with polycystic ovarian syndrome (PCOS): A meta-analysis of randomized clinical trials. Gynecol. Endocrinol..

[80] Sung C.T., Chao T., Lee A., Foulad D.P., Choi F., Juhasz M., Dobry A., Mesinkovska N.A. (2020). Oral Metformin for Treating Dermatological Diseases: A Systematic Review. J. Drugs Dermatol..

[81] Stanescu A.M.A., Simionescu A.A., Florea M., Diaconu C.C. (2021). Is Metformin a Possible Beneficial Treatment for Psoriasis? A Scoping Review. J. Pers. Med..

[82] Piskovatska V., Storey K.B., Vaiserman A.M., Lushchak O. (2020). The Use of Metformin to Increase the Human Healthspan. Adv. Exp. Med. Biol..

[83] Mohammed I., Hollenberg M.D., Ding H., Triggle C.R. (2021). A Critical Review of the Evidence That Metformin Is a Putative Anti-Aging Drug That Enhances Healthspan and Extends Lifespan. Front. Endocrinol..

[84] Piskovatska V., Stefanyshyn N., Storey K.B., Vaiserman A.M., Lushchak O. (2019). Metformin as a geroprotector: Experimental and clinical evidence. Biogerontology.

[85] European Medicines Agency (2019). New Add-On Treatment to Insulin for Treatment of Certain Patients with Type 1 Diabetes.

[86] Chini F., Pezzotti P., Orzella L., Borgia P., Guasticchi G. (2011). Can we use the pharmacy data to estimate the prevalence of chronic conditions? a comparison of multiple data sources. BMC Public Health.

[87] Maio V., Yuen E., Rabinowitz C., Louis D., Jimbo M., Donatini A., Mall S., Taroni F. (2005). Using pharmacy data to identify those with chronic conditions in Emilia Romagna, Italy. J. Health Serv. Res. Policy.

[88] Purkiss S.F., Keegel T., Vally H., Wollersheim D. (2020). A comparison of Australian chronic disease prevalence estimates using administrative pharmaceutical dispensing data with international and community survey data. Int. J. Popul. Data Sci..

[89] Slobbe L.C.J., Füssenich K., Wong A., Boshuizen H.C., Nielen M.M.J., Polder J.J., Feenstra T.L., van Oers H.A.M. (2019). Estimating disease prevalence from drug utilization data using the Random Forest algorithm. Eur. J. Public Health.

[90] Walckiers D., Sartor F. (1996). Results of an epidemiological study on drug-treated intraocular hypertension in Belgium. J. Clin. Epidemiol..

[91] Vaes B., Ruelens C., Saikali S., Smets A., Henrard S., Renard F., van den Akker M., Van Pottelbergh G., Goderis G., Van der Heyden J. (2018). Estimating the prevalence of diabetes mellitus and thyroid disorders using medication data in Flanders, Belgium. Eur. J. Public Health.

[92] Cohen R., Fontbonne A., Weitzman S., Eschwege E. (1990). Estimation of the prevalence of diabetes mellitus in Israel based on hypoglycemic drug supply and consumption. Diabete Metab..

[93] Purkiss S., Keegel T., Vally H., Wollersheim D. (2021). Estimates of drug treated diabetes incidence and prevalence using Australian administrative pharmaceutical data. Int. J. Popul. Data Sci..

